# Individual exposure to ambient PM_2.5_ and hospital admissions for COPD in 110 hospitals: a case-crossover study in Guangzhou, China

**DOI:** 10.1007/s11356-021-16539-x

**Published:** 2021-09-21

**Authors:** Jie-Qi Jin, Dong Han, Qi Tian, Zhao-Yue Chen, Yun-Shao Ye, Qiao-Xuan Lin, Chun-Quan Ou, Li Li

**Affiliations:** 1 National Clinical Research Center for Kidney Disease, State Key Laboratory of Organ Failure Research, Department of Biostatistics, Guangdong Provincial Key Laboratory of Tropical Disease Research, School of Public Health, Southern Medical University, Guangzhou, 510515 China; 2 The Third Affiliated Hospital of Southern Medical University, Guangzhou, 510630 China; 3 Guangzhou Health Technology Identification & Human Resources Assessment Center, Guangzhou, 510080 China

**Keywords:** PM_2.5_, Satellite, COPD, Hospitalization, Case-crossover design

## Abstract

Few studies have evaluated the short-term association between hospital admissions and individual exposure to ambient particulate matter (PM_2.5_). Particularly, no studies focused on hospital admissions for chronic obstructive pulmonary disease (COPD) at the individual level. We assessed the short-term effects of PM_2.5_ on hospitalization admissions for COPD in Guangzhou, China, during 2014–2015, based on satellite-derived estimates of ambient PM_2.5_ concentrations at a 1-km resolution near the residential address as individual-level exposure for each patient. Around 40,002 patients with COPD admitted to 110 hospitals were included in this study. A time-stratified case-crossover design with conditional logistic regression models was applied to assess the effects of PM_2.5_ based on a 1-km grid data of aerosol optical depth provided by the National Aeronautics and Space Administration on hospital admissions for COPD. Further, we performed stratified analyses by individual demographic characteristics and season of hospital admission. Around 10 μg/m^3^ increase in individual-level PM_2.5_ was associated with an increase of 1.6% (95% confidence interval [CI]: 0.6%, 2.7%) in hospitalization for COPD at a lag of 0–5 days. The impact of PM_2.5_ on hospitalization for COPD was greater significantly in males and patients admitted in summer. Our study strengthened the evidence for the adverse effect of PM_2.5_ based on satellite-based individual-level exposure data.

**Supplementary Information:**

The online version contains supplementary material available at 10.1007/s11356-021-16539-x.

## Introduction

Fine particulate matter with aerodynamic diameters < 2.5 μm (PM_2.5_) is a great concern for public health since it can penetrate into the lung deeply and induce respiratory diseases (Chen et al. 2017; Jiang et al. 2016; Kloog et al. 2012; Liu et al. 2018; Yang et al. 2020). Chronic obstructive pulmonary disease (COPD) is a major chronic respiratory disease, and the prevalence of COPD reached 251 million globally in 2016 (Hopke et al. 2019; Salimi et al. 2018; World Health Organization 2017). Elucidating the impact of exposure to PM_2.5_ on hospitalizations for COPD can help for developing interventions and thereafter reducing the burden of diseases.

An increasing number of studies have associated exposure to PM_2.5_ with hospital admissions for respiratory diseases, including COPD (Harrison and Yin 2000; Hopke et al. 2019; Kim et al. 2015; Lin et al. 2020). However, the estimates of the association between PM_2.5_ and hospital admissions were not always consistent (Belleudi et al. 2010; Kim et al. 2015; Kloog et al. 2014; Ko et al. 2007; Lin et al. 2020; Rice et al. 2015). Geographical variations in the estimates of associations suggested in prior studies may be due to the disparities in the level of air pollutant concentration, regional climates and socio-economical levels, and characteristics of local residents (Kumar et al. 2018). Thus, reports of different study locations can be beneficial for delineating a comprehensive contour of the adverse health effect of PM_2.5_.

More importantly, previous studies examined health effects of ambient PM_2.5_ exposure by using the PM_2.5_ level of the nearest station as individual exposure or more commonly using the average PM_2.5_ level of a few monitoring stations as a proxy of exposure for the whole population (Harrison and Yin 2000; Hopke et al. 2019; Kim et al. 2015; Lin et al. 2020; Santus et al. 2012; Zhang et al. 2019). The limited number of monitoring stations is not necessarily representative enough for the exposure level of the population in a location and thus there may be bias in such proxy (Goldman et al. 2011; Liu et al. 2017; Samet et al. 2000). In addition, the PM_2.5_ level calculated from selected monitoring stations ignores the spatial gradient of the exposure level among patients which may lead to misclassification of the exposure as well (Faustini et al. 2012; Liu et al. 2018; Moolgavkar and Suresh 2000; Tian et al. 2018). With the development of satellite monitoring technology, some models have been developed to estimate the level of ambient PM_2.5_ based on satellite data (Danesh et al. 2019; Guo et al. 2018; Lin et al. 2018). We previously proposed a method for a high-coverage and accurate estimation of daily PM_2.5_ at a 1-km resolution in mainland China based on the satellite data of atmospheric aerosol optical depth (AOD) by solving the problem of high missing rate, which is usually ignored in previous studies and leads to inaccurate estimation of PM_2.5_(Chen et al. 2020). The 1-km estimates of PM_2.5_ which can be matched with the residential addresses are expected to be a more precise proxy for the exposure level of PM_2.5_ for the subjects involved in studies on the health effect of air pollution, and the effect estimates tend to be more reliable (Hennig et al. 2020; Wong et al. 2015; Woo et al. 2020). Several cohort studies have applied satellite-derived exposure data to quantify the long-term effects of PM_2.5_ on health outcomes, with the data of addresses of subjects (Danesh et al. 2019; Guo et al. 2018; Lin et al. 2018). To our knowledge, there is no previous study assessing the short-term effects of PM_2.5_ on hospital admissions for COPD using satellite-based estimates of PM_2.5_ at a 1-km resolution or higher as individual exposure level, although Kloog et al. (2014) estimated the short-term effects on all-cause hospital admissions using PM_2.5_ at a 10 × 10-km spatial resolution in the Mid-Atlantic.

Guangzhou is one of the largest cities in the south of China (latitude: 23°07′N; longitude 113°15′E), covering an area of 7434.4 km^2^ with 14.9 million permanent residents. The average annual PM_2.5_ concentration in Guangzhou was from 37.0 to 46.8 μg/m^3^ between 2014 and 2015, which was much higher than the air quality guideline of WHO, 10 μg/m^3^ (World Health Organization 2005). The present study aims to assess the short-term effects of ambient PM_2.5_ on hospital admissions for COPD in Guangzhou, China, during 2014–2015, based on satellite-derived estimates of daily PM_2.5_ concentrations at a 1-km resolution near the residential address as individual-level exposure for each individual.

## Materials and methods

### Data sources

The data on the home page of electronic medical records for all individual hospitalization due to COPD admitted in 110 hospitals during 2014–2015 in Guangzhou were retrieved from the Guangzhou Health Information Center, which is a part of the National Health Statistics Network Direct Report system having standard operation procedures and sophisticated measures for quality control (National Health and Family Planning Commission of the People's Republic of China 2007). The database of this system of Guangzhou encompassed all 122 hospitals ranked level II and above, in which 110 hospitals admitting COPD patients were used in this research. Principal diagnosis was coded according to the International Statistical Classification of Diseases and Related Health Problems, 10th Revision (ICD-10). We extracted the information for all individuals admitted due to COPD (ICD-10 code: J40-J44), including sex, age, residential address, occupational class, marital status, and date of admission. Daily meteorological data, including daily mean temperature and relative humidity, were downloaded from the National Meteorological Center (http://data.cma.cn/).

The study was approved by the ethical committee of Southern Medical University, and patient informed consent was waived since only de-identified data derived from the official health information system were provided and analyzed anonymously.

### Exposure assessment

In our previous study, we applied extreme gradient boosted (XGBoost) imputation to fill the missing gap of a 1-km multiangle implementation of AOD obtained from the National Aeronautics and Space Administration, and the coverage of AOD was significantly increased from 15.46 to 98.64% on average in mainland China (Chen et al. 2020). Then a combined method of non-linearexposure-lag-response model and XGBoost was applied to predict daily 1 × 1-km PM_2.5_ concentrations using meteorological variables and the XGBoost-interpolated AOD, with high predictive accuracy (cross-validation *R*^2^ = 0.81 for Guangzhou) (Chen et al. 2020). Daily levels of PM_2.5_ were well estimated for all grids including those without monitoring stations. Next, each patient’s residential address was matched to the 1-km grid cell according to the longitude and latitude and then the corresponding daily PM_2.5_ concentrations on the current day of admission and the previous 6 days before admission in the grid cell were treated as the proxy of daily exposure for the patient.

### Statistical analysis

We adopted a time-stratified case-crossover design to assess the associations between PM_2.5_ and hospital admissions for COPD. This approach has been widely applied to evaluate the health effects of air pollution in which each case serves as his/her own control (Levy et al. 2001; Szyszkowicz et al. 2018; Tsai et al. 2013). In this study, the case days were defined as the dates of admission, while the referent days were those that fell on the same day of the week within the same month as the case day. Since the case period and the referent period are very close in time, this design avoids the influence of individual characteristics and long-term and seasonal trends on the effect estimation (Bateson and Schwartz 1999; Janes et al. 2005).

We used conditional logistic regression models to quantify the impacts of PM_2.5_ on hospitalizations due to COPD. The model was of the following form:

$$ logit\left({P}_i\right)={\alpha}_i+ NS\left({T}_{06}, df=3\right)+ NS\left({RH}_{06}, df=3\right)+\delta Holiday+\beta {PM}_{2.5} $$

*P*_*i*_ and *α*_*i*_ are the probability of hospitalization and the intercept for stratum *i*. According to previous studies, a natural cubic spline (*NS*) with three degrees of freedom (*df*s) was applied for the moving average of temperature (*T*) and relative humidity (*RH*) at a lag of 0–6 days (Liu et al. 2017; Lu et al. 2019; Tian et al. 2018). In addition, an indicator variable of public holidays (Holiday) was also included (Hwang et al. 2017; Ma et al. 2019). *δ* is the regression coefficient for Holiday. Here, we considered the effects of PM_2.5_ on hospitalizations for COPD at lags up to 6 days. Both single-day and multiple-day lags were considered in this study (Devries et al. 2016; Hwang et al. 2017; Liu et al. 2018).

Next, we conducted stratified analysis by sex, age (<65 and ≥65 years), occupational class (the unemployed, blue-collar workers, and white-collar workers), marital status (married, unmarried, and divorced/widowed), and season of hospital admission (winter [December to February], spring [March to May], summer [June to August], and autumn [September to November]) (Ma et al. 2019). The differences in the effects of PM_2.5_ on hospital admissions for COPD between two subgroups were tested via the *Z* test with the following formula:

$$ Z=\frac{\beta_1-{\beta}_2}{\sqrt{SE{\left({\beta}_1\right)}^2+ SE{\left({\beta}_2\right)}^2}} $$

where *β*_1_ and *β*_2_ represent the regression coefficients of PM_2.5_ for two subgroups and *SE*(*β*_1_) and *SE*(*β*_2_) are the corresponding standard errors. Sensitivity analyses were conducted to assess the robustness of the results by changing *df*s for temperature and relative humidity from 3 to 2–6 and using different lag days for meteorology with lag 0–14 and lag 0–21.

All statistical tests were two-sided, and *P* < 0.05 was considered statistically significant. We performed all data analyses using R software (version 3.5.1, R Foundation for Statistical Computing).

## Results

**Fig. 1:**
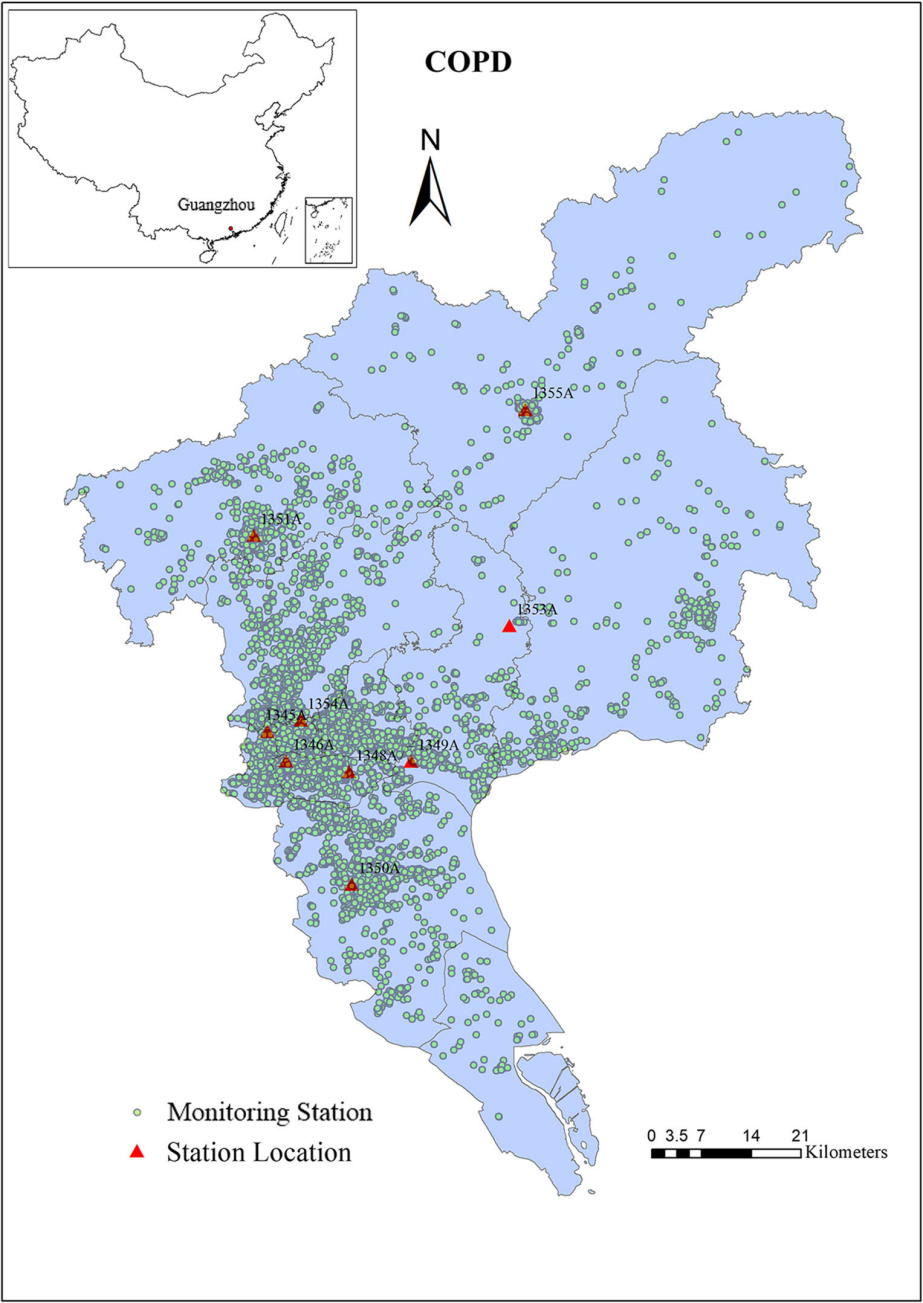
Locations of monitoring stations of PM_2.5_ and places of residence of the included patients admitted with chronic obstructive pulmonary disease

A total of 40,002 patients admitted to 110 hospitals with COPD from 2014 to 2015 were included in the analysis (Fig. 1). Patients hospitalized for COPD were predominately males (74.1%) and those aged ≥65 years (83.8%) (Table 1). The unemployed and married individuals accounted for 81.5% and 93.3% of the hospital admissions for COPD. It seemed that the differences in the proportions of hospitalizations for COPD across four seasons were small (Table 1). Average satellite-derived1-km-resolution PM_2.5_ concentrations during the study period were 42.6 μg/m^3^ (ranges from 6.0 to 164.4 μg/m^3^) for COPD patients. Daily temperature and relative humidity were on average 22.2°C (ranges from 5.2 to 31.4°C) and 79.8% (ranges from 31.5 to 97.5%) during the study period (Table 2).

**Table 1:** Characteristics of included patients admitted with chronic obstructive pulmonary disease

Variable	Group	*N* (%)
Sex	Male	29,652 (74.1)
	Female	10,350 (25.9)
Age	<65 years	6,497 (16.2)
	≥65 years	33,505 (83.8)
Occupational class	Unemployed	22,509 (81.5)
	White collar	1,170 (4.2)
	Blue collar	3,948 (14.3)
Marital status	Married	36,671 (93.3)
	Unmarried	1,668 (4.2)
	Divorce/widowed	969 (2.5)
Season	Winter	9,749 (24.4)
	Spring	11,306 (28.3)
	Summer	10,088 (25.2)
	Autumn	8,859 (22.1)

**Table 2:** Summary statistics of exposure level of satellite-based PM_2.5_ and meteorological conditions for patients with chronic obstructive pulmonary disease in Guangzhou, China, 2014–2015

Variable	Minimum	Median	Maximum	Mean	SD
PM_2.5_ (mg/m^3^)	6.0	38.7	164.4	42.6	21.0
Temperature (°C)	5.2	23.7	31.4	22.2	6.1
Relative humidity (%)	31.5	80.5	97.5	79.8	9.9

Abbreviations: *COPD*, chronic obstructive pulmonary disease; *SD*: standard deviation; *T*, temperature; *RH*, relative humidity

The effect of PM_2.5_ on hospital admission for COPD was higher on the current day in the single-day model, meanwhile the effect was the greatest for the moving average of 0–5 days than for other lagged days (Table 3). It was estimated that a 10-μg/m^3^ increase in PM_2.5_ was associated with an increase of 1.6% (OR = 1.016, 95% confidence interval [CI]: 1.006, 1.027) in hospitalization for COPD at a lag of 0–5 days.

**Table 3:** Odds ratios of hospital admissions for chronic obstructive pulmonary disease per 10-μg/m^3^ increase in PM_2.5_ at different lag days

Lag (days)	OR (95% CI)	*P* value
0	1.012 (1.004, 1.019)	0.002
1	1.007 (1.000, 1.014)	0.056
2	1.006 (0.999, 1.013)	0.097
3	1.006 (0.999, 1.013)	0.115
4	1.008 (1.001, 1.015)	0.027
5	1.006 (0.999, 1.013)	0.100
6	1.001 (0.994, 1.007)	0.873
0–1	1.012 (1.004, 1.021)	0.004
0–2	1.013 (1.004, 1.022)	0.005
0–3	1.014 (1.004, 1.024)	0.005
0–4	1.016 (1.005, 1.026)	0.003
0–5	1.016 (1.006, 1.027)	0.002
0–6	1.015 (1.004, 1.027)	0.006

Abbreviations: *OR*, odds ratio; 95% *CI*, 95% confidence interval

**Fig. 2:**
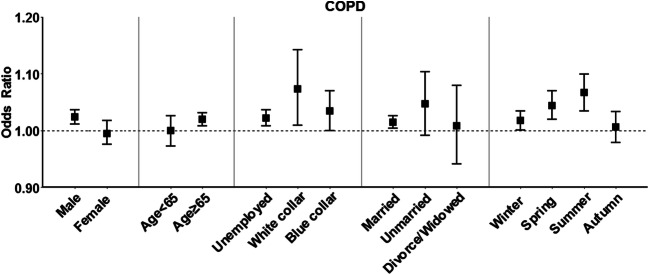
Odds ratios of hospitalizations for chronic obstructive pulmonary disease (COPD) per 10-μg/m^3^ increase of individual exposure level of PM_2.5_ at a lag of 0–5 days in subgroup analysis. Points and lines are point estimates and the corresponding 95% confidence interval of odds ratios. The dashed line indicates odds ratio = 1

We estimated the ORs of hospitalizations for COPD per 10-μg/m^3^ increase in individual exposure level of PM_2.5_, at lag 0–5 days, for different subgroups. The effect of PM_2.5_ on hospital admission for COPD was statistically significant for males (OR: 1.023, 95% CI: 1.011, 1.036), while the effect was statistically non-significant for females, and the difference in the effects of PM_2.5_ was statistically significant by sex (*P* = 0.046). It was witnessed that hospitalizations for COPD (OR: 1.020, 95% CI: 1.008, 1.032) increased with PM_2.5_ among people ≥65 years of age. However, the difference in the effects of PM_2.5_ was not statistically significant by age (*P* > 0.05). The effect of PM_2.5_ on hospitalizations for COPD seemed not to vary by occupational class and marital status (*P* > 0.05). A larger OR (1.052 [95% CI: 1.021, 1.084]) was estimated for patients admitted due to COPD in summer than in other seasons (*P* = 0.048) (Fig. 2 and Table S1).

According to the results of sensitivity analysis, the effect estimates were not substantially changed when changing the *df*s or lag days for temperature and relative humidity from 3 to 2–6(Table S2 and Table S3).

## Discussion

We estimated the short-term effect of ambient PM_2.5_ on hospital admissions for COPD based on satellite-derived1-km-resolution estimates of PM_2.5_ in Guangzhou, China. We found a significant association between hospital admissions for COPD with the ambient PM_2.5_ at individual exposure level, and the effect of PM_2.5_ was stronger in males and patients admitted during the warm season. Our findings provided more solid evidence for the association between ambient PM_2.5_ and hospital admissions for COPD with individual exposure data.

In this study, we estimated that a 10-μg/m^3^ increase in PM_2.5_ was associated with an increase of 1.6% in hospitalization for COPD at a lag of 0–5 days. Statistically significant associations between PM_2.5_ and hospital admissions for COPD were also reported in other studies using ground-level monitoring data of PM_2.5_, but the magnitude of estimates varied across study locations. For example, the corresponding effect estimates for COPD admissions were 2.06% at lag 0–6 days in Ningbo, China (Zhang et al. 2019) and 3.10% at lag 0–2 days in Milan, Italy (Santus et al. 2012). Lin et al. (2020) showed that the effect of PM_2.5_ on hospital admissions for COPD was highest at a distributed lag of 0–7 days (relative risks = 1.073, 95% CI: 1.016, 1.133) in Yinzhou District, China. Inconsistency was further observed in studies which reported statistically non-significant associations of PM_2.5_ with hospital admissions for COPD in some other cities (Belleudi et al. 2010; Liu et al. 2018; Peel et al. 2005; Slaughter et al. 2005; Stieb et al. 2009). A certain number of previous studies examined the associations with the data of a single or a few hospitals in which the sample size may be insufficient for the inference, and therefore, non-significant results were obtained (Peel et al. 2005; Slaughter et al. 2005). Previous studies on short-term associations between exposure to air pollutants and health outcomes commonly used the average measurements obtained from a few monitoring stations as the proxy of exposure (Lin et al. 2020; Santus et al. 2012; Tian et al. 2018; Zuo et al. 2019). Measurements of air pollutant concentrations obtained from monitoring stations can capture the temporal variation of the average exposure level. However, using the average measurements from a limited number of monitoring stations cannot fully reflect the spatial variation in exposure level of subjects which may lead to bias in the estimates of associations between air pollutants and health outcomes due to the measurement error of exposure (Hwang et al. 2017; Liu et al. 2017; Tian et al. 2018; Xie et al. 2019; Zuo et al. 2019). On this issue, an accurate proxy of individual exposure using satellite-based PM_2.5_ with high resolution is crucial when investigating the health effects of air pollution. In addition, different levels of air pollutant concentrations, demographic characteristics and healthcare facilities, and the criteria for hospital admission may explain the variations in estimates of the associations between PM_2.5_ and hospital admissions for COPD (Kumar et al. 2018). The plausible mechanism that PM_2.5_ could affect COPD was the airway irritation leading to interactions with the immune system to cause respiratory infections, oxidative stress, and pulmonary inflammation (Mu et al. 2014; Szyszkowicz et al. 2018). In addition, the toxicity and irritability of particulate matter may aggravate lung infection, reducing the immune efficacy of the lung (Tsai et al. 2013).

Exploring the potential effect modifiers of PM_2.5_ on hospital admissions can be helpful for identifying potentially susceptible populations and for developing a more accurately targeted intervention. The differences in the effects of PM_2.5_ on hospital admissions for COPD by sex were statistically significant in our study, which was in accordance with prior studies (Lin et al. 2018). A higher effect was observed in males, and the difference between gender may be due to lifestyle behaviors (tobacco and alcohol consumption, exercise, and diet) (Xie et al. 2019). As for the disparities by age, we found that the effects of PM_2.5_ on hospitalizations were statistically significant in individuals ≥65 years of age for COPD, while non-significant results were observed in those <65 years. Such finding was also observed in a study which explored the association between PM_2.5_ and hospital admissions for acute exacerbation of COPD in southwestern Taiwan, China (Hwang et al. 2017). A study in Hefei, China, claimed that the effect of PM_2.5_ on COPD hospitalization differed by age, although the statistical significance of the difference was not examined (Xie et al. 2019). Regarding the disparities in the associations of PM_2.5_ with hospitalizations for COPD by occupational class and marital status, a relatively small sample size for some subgroups in the present study may account for the lack of statistical power to detect significant effects.

We found that the effects of PM_2.5_ on hospitalizations for COPD varied by season, with the highest estimates of the effects occurring in summer. The variation in the effects of PM_2.5_ by season could be due to that people tend to participate in outdoor activities and may open windows more frequently during warm months, leading to more exposure to ambient air pollutants (Stafoggia et al. 2016; Tian et al. 2018). In the context of global warming, the temperature is expected to increase, and the heat wave will occur more frequently (Lee et al. 2018; Yang et al. 2021; Zhang et al. 2017). Consequently, adverse health impacts of exposure to PM_2.5_ should be paid more attention to in the future given the higher effects of PM_2.5_ on hospitalizations for COPD observed in summer. Further studies are required to clarify the underlying mechanism of the variation in effects of PM_2.5_ by season and then inform better preparedness for the potentially elevated burden associated with PM_2.5_ in the future.

The current study was subject to several limitations. First, we did not assess the effects of exposure to other air pollutants on hospitalizations for COPD since the individual exposure data tailored for each patient were not available. Second, the sample sizes were relatively small for some subgroups which may be insufficient for the inference of the effects of PM_2.5_ for these subgroups. Third, the current study controlled for the effects of temperature, relative humidity, and holiday on hospitalizations for COPD, but other potentially influential factors, such as respiratory virus activity, were not considered because of the unavailability of data. Fourth, although compared with the PM_2.5_ averaged from the monitoring stations, the satellite-based PM_2.5_ is more accurately reflected on the spatial gradient at a 1 × 1-km solution, this address-level exposure estimation is still a proxy of individual exposure to outdoor PM_2.5_, and we cannot rule out the residual measurement error. Further studies can be conducted to achieve more accurate estimates of the effects of PM_2.5_ with the use of personal exposure data accounting for both outdoor and indoor exposure levels.

## Conclusions

We found a significant association between hospital admissions for COPD with the ambient PM_2.5_, and our study strengthened the evidence for the adverse effect of PM_2.5_ based on satellite-based individual-level exposure data. The impact of PM_2.5_ on hospitalization for COPD was greater in males and patients admitted in summer.

## Supplementary Information

ESM 1 (DOCX 39 kb)

## Data Availability

The datasets used and/or analyzed during the current study are available from the corresponding author on reasonable request.
