# Supplementary material for: Individual exposure to ambient PM2.5 and hospital admissions for COPD in 110 hospitals: a case-crossover study in Guangzhou, China
Source: Environ Sci Pollut Res Int. 2021 Sep 21;29(8):11699–706. doi: 10.1007/s11356-021-16539-x (PMC8794997; doi:10.1007/s11356-021-16539-x)
Supplement: Supplementary file 1 — (DOCX 39 kb) [file 11356_2021_16539_MOESM1_ESM.docx]

**Supplemental Materials**

**Individual exposure to ambient PM_2.5_ and hospital admissions for COPD in 110 hospitals: a case-crossover study in Guangzhou, China** Jie-Qi Jin^a,#^, Dong Han^b,a,#^, Qi Tian^c,^**^#^**, Zhao-Yue Chen^a,^**^#^**, Yun-Shao Ye^c^, Qiao-Xuan Lin^c^, Chun-Quan Ou^a^, Li Li^a,*^

**^a^**National Clinical Research Center for Kidney Disease, State Key Laboratory of Organ Failure Research, Department of Biostatistics, Guangdong Provincial Key Laboratory of Tropical Disease Research, School of Public Health, Southern Medical University, Guangzhou 510515, China

**^b^**The Third affiliated Hospital of Southern Medical University, Guangzhou 510630, China

**^c^**Guangzhou Health Technology Identification & Human Resources Assessment Center, Guangzhou 510080, China

*^#^* These authors contributed equally to this work.

^*^Corresponding author:

L. Li (lylygdsg@163.com)

State Key Laboratory of Organ Failure Research, Department of Biostatistics, Guangdong Provincial Key Laboratory of Tropical Disease Research, School of Public Health, Southern Medical University, Guangzhou, China

Phone: (8620) 61360456

Fax: (8620) 61648319

**OUTLINE**

| **Title** | **Page** |
| --- | --- |
| **Table S1.** Estimates of odds ratios (ORs) of hospital admissions for COPD per 10 *μg/m*^3^ increase in PM_2.5_ at lag 0-5 days in the stratified analysis. | **1-2** |
| **Table S2.** Estimates of odds ratios (ORs) of hospital admissions for COPD per 10 *μg/m*^3^ increase in PM_2.5_ at lag 0-5 days in the sensitivity analysis, with different degrees of freedom (*df*s) for temperature and relative humidity. | **3** |
| **Table S3.** Estimates of odds ratios (ORs) of hospital admissions for COPD per 10 *μg/m*^3^ increase in PM_2.5_ at lag 0-5 and days in the sensitivity analysis, with different lags for temperature and relative humidity. | **4** |

**Table S1.** Estimates of odds ratios (ORs) of hospital admissions for COPD per 10 *μg/m*^3^ increase in PM_2.5_ at lag 0-5 days in the stratified analysis.

| Variable | OR (95% CI) | *P*-value |
| --- | --- | --- |
| Sex | | |
| Male | 1.023 (1.011, 1.036) * | Ref |
| Female | 0.998 (0.977, 1.019) | 0.046 |
| Age | | |
| <65 years | 0.998 (0.972, 1.025) | Ref |
| ≥65 years | 1.020 (1.008, 1.032) * | 0.149 |
| Occupational class | | |
| Unemployed | 1.021 (1.007, 1.036) * | Ref |
| White Collar | 1.080 (1.015, 1.150) * | 0.087 |
| Blue Collar | 1.043 (1.008, 1.079) * | 0.253 |
| Marital Status | | |
| Married | 1.016 (1.005, 1.027) * | Ref |
| Unmarried | 1.023 (0.971, 1.078) | 0.800 |
| Divorce/Widowed | 1.011 (0.944, 1.083) | 0.888 |
| Season | | |
| Winter | 1.015 (1.000, 1.030) * | Ref |
| Spring | 1.045 (1.020, 1.070) * | 0.096 |
| Summer | 1.052 (1.021, 1.084) * | 0.048 |
| Autumn | 1.005 (0.977, 1.034) | 0.352 |

Abbreviations: 95% CI, 95% confidence interval; Ref, referent group.

^*^ *P*-value<0.05

**Table S2.** Estimates of odds ratios (ORs) of hospital admissions for COPD per 10 *μg/m*^3^ increase in PM_2.5_ at lag 0-5 and days in the sensitivity analysis, with different degrees of freedom (*df*s) for temperature and relative humidity.

| Variable | df | OR (95% CI) | *P*-value |
| --- | --- | --- | --- |
| Temperature | 2 | 1.016 (1.006, 1.027) | 0.003 |
|  | 3^a^ | 1.016 (1.006, 1.027) | 0.002 |
|  | 4 | 1.017 (1.006, 1.027) | 0.002 |
|  | 5 | 1.017 (1.007, 1.028) | 0.001 |
|  | 6 | 1.020 (1.009, 1.031) | <0.001 |
| Relative humidity | 2 | 1.018 (1.007, 1.029) | 0.001 |
|  | 3^a^ | 1.016 (1.006, 1.027) | 0.002 |
|  | 4 | 1.020 (1.009, 1.031) | <0.001 |
|  | 5 | 1.020 (1.009, 1.031) | <0.001 |
|  | 6 | 1.021 (1.010, 1.032) | <0.001 |

Abbreviations: 95% CI, 95% confidence interval.

^a^ The *df* used in the main analysis.

**Table S3.** Estimates of odds ratios (ORs) of hospital admissions for COPD per 10 *μg/m*^3^ increase in PM_2.5_ at lag 0-5 and days in the sensitivity analysis, with different lags for temperature and relative humidity.

| Variable | Lag(days) | OR (95% CI) | *P*-value |
| --- | --- | --- | --- |
| Temperature | 0-6^a^ | 1.016 (1.006, 1.027) | 0.003 |
|  | 0-14 | 1.013 (1.004, 1.022) | 0.005 |
|  | 0-21 | 1.011 (1.001, 1.020) | 0.035 |
| Relative humidity | 0-6^a^ | 1.016 (1.006, 1.027) | 0.003 |
|  | 0-14 | 1.011 (1.000, 1.023) | 0.046 |
|  | 0-21 | 1.015 (1.005, 1.025) | 0.004 |

Abbreviations: 95% CI, 95% confidence interval.

^a^ The *df* used in the main analysis.
